# Swallowing function after transoral surgery for laryngopharyngeal cancer

**DOI:** 10.1371/journal.pone.0270509

**Published:** 2022-06-24

**Authors:** Tsutomu Ueda, Kouhei Yumii, Yuji Urabe, Nobuyuki Chikuie, Takayuki Taruya, Takashi Kono, Takao Hamamoto, Masaya Takumida, Minoru Hattori, Takashi Ishino, Sachio Takeno

**Affiliations:** 1 Department of Otorhinolaryngology, Head and Neck Surgery, Graduate School of Biomedical and Health Sciences, Hiroshima University, Hiroshima, Japan; 2 Department of Medicine and Molecular Science, Division of Frontier Medical Science, Programs for Biomedical Research, Graduate School of Biomedical Sciences, Hiroshima University, Hiroshima, Japan; 3 Center for Medical Education Institute of Biomedical & Health Sciences, Hiroshima University, Hiroshima, Japan; King Faisal Specialist Hospital and Research Center, SAUDI ARABIA

## Abstract

Transoral surgery (TOS) has been widely used to treat laryngopharyngeal cancers. Although TOS is a minimally invasive procedure, postoperative complications, such as postoperative dysphagia, may occur, which can lead to a poor quality of life for patients undergoing TOS. This study aimed to investigate factors that may affect swallowing function in patients who underwent TOS for laryngopharyngeal cancers. Swallowing function of 84 patients who underwent endoscopic resection for oropharyngeal, hypopharyngeal, and supraglottic lesions was evaluated by the Functional Outcome Swallowing Scale, and predictors for postoperative dysphagia were identified. Multivariate analysis identified the following factors as independent predictors for postoperative dysphagia: Eastern Cooperative Oncology Group Performance Status (ECOG PS, p = 0.008), prior neck radiation therapy (p = 0.008), and operative time (p = 0.021). This study suggests that patients with poor ECOG PS or those who received prior neck radiation therapy should be fully assessed for preoperative swallowing function. In the future, we would like to clarify the criteria for preoperative swallowing evaluation to create a system that can identify patients suitable for TOS.

## Introduction

Early-stage laryngopharyngeal cancers have been detected frequently with widespread use of narrow-band imaging (NBI) by magnifying endoscopy [1,2]. These diseases are treated with surgery, radiotherapy, or chemoradiotherapy. Severe dysphagia may reportedly develop after radiation-based therapy for laryngopharyngeal cancers despite functional organ preservation [3].

Transoral surgery (TOS) has been reported as useful for resecting Tis, T1, T2, and selected T3 stages of laryngopharyngeal cancer [4]. Moreover, it has been recently reported that TOS can resect lesions that underwent treatment failure after definitive radiation therapy (RT) or those that develop additional primary lesions within the RT field [5].

Although TOS is a minimally invasive procedure, postoperative complications may still occur.

Postoperative dysphagia can occur after TOS and can significantly impair a patient’s quality of life.

In our facility, Tis, T1, T2, and T3 stages of laryngopharyngeal cancer have been treated with a TOS technique called endoscopic laryngopharyngeal surgery (ELPS) since 2007. ELPS is performed for recurrence after radiotherapy and additional primary lesions within the RT field.

This study aimed to investigate the factors that may influence swallowing function in patients who underwent TOS for laryngopharyngeal cancers and to clarify the adaptation of TOS. The goal was to eliminate postoperative dysphagia.

## Methods

We conducted a retrospective cohort study in patients with oropharyngeal, hypopharyngeal, and supraglottic lesions who underwent endoscopic resection at Hiroshima University Hospital in Japan between August 2009 and May 2020. Eligible patients were aged ≥18 years at the time of resection and had an Eastern Cooperative Oncology Group Performance Status (ECOG PS) of 0–1. Patients who were able to receive oral intake, had normal swallowing function, and were asymptomatic before surgery were included. This study was approved by the institutional review board of Hiroshima University Hospital (Hiroshima University Hospital IRB E-2039), in accordance with the Declaration of Helsinki ethical principles for medical research involving human participants. The study protocol was posted at our institution, and all patients were offered the opportunity to opt out of the study. We applied the opt-out method to obtain consent on this study using the poster. The poster was approved by the Institutional Review Board.

Endoscopic resection involves endoscopic mucosal resection with endoscopic submucosal dissection (ESD) or endoscopic laryngopharyngeal surgery (ELPS) using a curved laryngoscope. ELPS is a hybrid of head and neck surgery and gastrointestinal endoscopic treatment. The concept is the same as that of ESD in that both involve *en bloc* resection of a cancer lesion following submucosal injection. The main difference is that the procedure is performed by a head and neck surgeon using both hands in ELPS [6].

We retrospectively collected patient data on age, sex, weight, height, body mass index (BMI), primary tumor location, ECOG PS, smoking history, alcohol consumption, history of head and neck/esophageal cancers, prior neck RT, hemogram parameters (white blood cell [WBC] count, absolute neutrophil count [ANC], absolute lymphocyte count [ALC], hemoglobin [Hb] level, platelet count [Plt], albumin level [Alb], neutrophil-to-lymphocyte ratio [NLR], and platelet-to-lymphocyte ratio [PLR]), prognostic nutritional index (PNI), pathological results, tumor size, operative time, period from discovery to surgery, and operative result from electronic medical records. According to the Asia-Pacific BMI classifications [7], patients with BMI of <18.5 kg/m^2^, ≥18.5 and <25 kg/m^2^, ≥25 kg/m^2^ were classified as underweight, normal range, and obese, respectively.

Serum albumin value was measured by the bromcresol purple (BCP) improved method, and the normal range is defined as 4.1–5.1 g/dL by our institutional criteria. A diagnosis of anemia was based on a hemoglobin level of <13.7 g/dL in male and <11.6 g/dL in females, and hypoalbuminemia was defined as a serum albumin level of <3.5 g/dL. The PNI was calculated as 10 × albumin + 0.005 × ALC.

Swallowing function was evaluated using the Functional Outcome Swallowing Scale (FOSS) at 6 months after surgery. The FOSS categorizes swallowing function into six stages as follows: stage 0 = normal function and asymptomatic; stage 1 = normal function with episodic or daily symptoms of dysphagia; stage 2 = compensated abnormal function manifested by significant dietary modifications or prolonged mealtime without weight loss or aspiration; stage 3 = decompensated abnormal function with weight loss of ≤10% of body weight over 6 months due to dysphagia, daily cough, gagging, or aspiration during meals; stage 4 = severely decompensated abnormal function with weight loss of >10% of body weight over 6 months due to dysphagia or severe aspiration with bronchopulmonary complications, non-oral feeding is recommended for nutrition; and stage 5 = non-oral feeding for all nutrition. Hoarseness was evaluated using the G component (overall dysphonia) of the grade (G), roughness (R), breathiness (B), asthenia (A), strain (S) scale on a 4-point scale as follows: G0, normal; G1, mild; G2, moderate; and G3, severe, diagnosed by a speech pathologist more than 3 months after surgery.

### Data analysis

Continuous variables are reported as medians and ranges and were analyzed using the non-parametric Mann–Whitney U test. Categorical variables are reported as numbers (%) and were analyzed using Fisher’s exact test. Patient characteristics were compared between those with FOSS stage 0 and FOSS stages 1–5. Multiple logistic regression analysis was used to evaluate the association between the FOSS stage and other clinicopathological factors. Variables that showed statistically significant associations in univariate analysis were entered into a multivariate logistic regression model. Differences between the results of the comparative tests were considered statistically significant at a two-tailed p-value threshold of <0.05. Receiver operating characteristic (ROC) curves were calculated to determine the optimal cut-off value and area under the ROC curve of NLR, PLR, PNI, tumor size, and operation time. Calculations were performed using SPSS (version 27; IBM, Armonk, NY, USA).

## Results

Patient characteristics are listed in Table 1. The median age at treatment was 65 years (range, 40–87 years), and 92.9% of the patients were male. The median BMI was 21.2 kg/m^2^ (range, 13.96–30.16 kg/m^2^). Moreover, 17 patients were underweight, 55 had normal range and 12 were obese. Thereafter, we focused on low BMI, dividing the study into underweight and others.

**Table 1:** Clinicopathological characteristics.

Variables		
**N**		84
**Age (years)**	Median (range)	65 (40–87)
**Sex**	Male	78 (92.9%)
	Female	6 (7.1%)
**BMI (kg/m** ^**2**^ **)**	Median (range)	21.2 (13.96–30.16)
**Primary site**		
**Oropharynx**	Lateral wall	2 (11.1%)
	Posterior wall	9 (50.0%)
	Anterior wall	6 (33.3%)
	Superior wall	1 (5.6%)
**Hypopharynx**	Pyriform sinus	51 (82.3%)
	Posterior wall	7 (11.3%)
	Post-cricoid	4 (6.5%)
**Larynx**	Supraglottic	4
**ECOG PS**	0	75 (89.3%)
	1	9 (10.7%)
**Smoking habit**	Never	8 (9.5%)
	Former	53 (63.1%)
	Smokers	23 (27.4%)
**Alcohol drinking**	Never	3 (3.6%)
	Former	14 (16.7%)
	Social drinkers	11 (13.1%)
	Drinkers	56 (66.7%)
**History of head and neck cancer**	Yes	57 (67.0%)
	No	27 (32.1%)
**History of esophageal cancer**	Yes	66 (78.6%)
	No	18 (21.4%)
**Prior neck radiation therapy**	Yes	21 (25.0%)
	No	63 (75.0%)
**WBC (cells/μL)**	Median (range)	5115 (2080–12950)
**ANC (cells/μL)**	Median (range)	3075 (950–9360)
**ALC (cells/μL)**	Median (range)	1340 (400.0–3110.0)
**Hemoglobin (g/dL)**	Median (range)	13.2 (10.2–16.8)
**Platelets (×10**^**3**^ **cells/μL)**	Median (range)	21.8 (6.6–41.7)
**Albumin (g/dL)**	Median (range)	4.20 (2.9–5.2)
**Neutrophil/lymphocyte ratio**	Median (range)	2.23 (0.64–6.08)
**Platelet/lymphocyte ratio**	Median (range)	162 1 (32.7–472.5)
**Prognostic nutritional index**	Median (range)	48.4 (33.6–63.9)
**Histology**	*In situ*	40 (47.6%)
	SCC	44 (52.4%)
**pT**	pTis	40 (47.6%)
	pT1	33 (39.3%)
	pT2	10(11.9%)
	pT3	1 (1.2%)
**Lymphovascular invasion**	Negative	82 (97.6%)
	Positive	2 (2.4%)
**Blood vessel invasion**	Negative	77 (91.7%)
	Positive	7 (8.3%)
**Period from discovery to surgery (days)**	Median (range)	72(0–998)
**Tumor size (mm** ^**2**^ **)**	Median (range)	400 (14.0–2500.0)
**Operative time (min)**	Median (range)	81(13–600)
**FOSS**	Stage 0	53 (63.1%)
	Stage 1	15 (17.9%)
	Stage 2	8 (9.5%)
	Stage 3	4 (4.8%)
	Stage 4	2 (2.4%)
	Stage 5	2 (2.4%)

BMI, body mass index; ECOG PS, Eastern Cooperative Oncology Group Performance Score; WBC, white blood cells; ANC, absolute neutrophil count; ALC, absolute lymphocyte count; FOSS, Functional Outcome Swallowing Scale.

There were 18 (21.4%) lesions in the oropharynx, 62 (73.8%) in the hypopharynx, and 4 (4.8%) in the larynx. In the oropharynx, two (11.1%) lesions were in the lateral wall, nine (50.0%) in the posterior wall, six (33.3%) in the anterior wall, and one (5.6%) in the superior wall. In the hypopharynx, 51 (82.3%) lesions were in the piriform sinus, 7 (11.3%) in the posterior wall, and 4 (6.5%) in the post-cricoid region. In the larynx, four lesions were in the supraglottic area.

Eight patients (9.5%) were never-smokers, 53 (63.1%) were former smokers, and 23 (27.4%) were current smokers. Three patients (3.6%) did not consume alcohol, 14 (16.7%) were former drinkers, 11 (13.1%) were social drinkers, and 56 (66.7%) consumed alcohol regularly every day.

Seventy-five patients (89.3%) had ECOG PS 0, and nine patients had ECOG PS 1. Fifty-seven patients (67.0%) had a history of head and neck cancers and 66 (78.6%) had a synchronous or previous history of esophageal cancer. All cancers of the esophagus and head and neck region were treated with methods, such as endoscopic resection, (chemo)radiotherapy, and surgery. Most of the patients were identified as having cancer by follow-up examinations for esophageal or head and neck cancers. Twenty-one (25.0%) patients had a history of neck irradiation and 63 (75.0%) had no exposure to radiation.

The median WBC count, ANC, ALC, Hb, Plt, and Alb were 5115/μL, 3075/μL, 1340/μL, 13.2 g/dL, 21.8×10^3^ cells/μL, and 4.2 g/dL, respectively. The median NLR, PLR, and PNI were 2.23, 162.1, and 48.4, respectively.

All resected specimens were squamous cell carcinoma (SCC). Forty (47.6%) lesions were carcinoma *in situ* and 44 (52.4%) lesions invaded the subepithelium. There were 40 Tis lesions (57.4%), 33 T1 lesions (39.3%), 10 T2 lesions (11.9%), and 1 T3 lesion (1.2%).

Two lesions had lymphovascular invasion and seven lesions had blood vessel invasion.

The median tumor size was 400 mm^2^ (range, 14–2500 mm^2^) and the median operative time was 81 min (range, 13–600 min). The median time from the discovery of the lesion to surgery was 72 days (range, 0–998 days).

At 6 months after surgery, 53 patients (63.1%) were FOSS stage 0, 15 (17.9%) were stage 1, 8 (9.5%) were stage 2, 4 (4.8%) stage 3, 2 (2.4%) were stage 4, and 2 (2.4%) were stage 5. The cut-off values from ROC curves for low and high NLP (<2.1, ≥2.1), low and high PLR (<237, ≥237), low and high PNI (<44.5, ≥44.5), low and high tumor size (≤323, >323), and low and high operative time (≤103, >103) were determined.

Univariate analysis showed that age (p = 0.009), smoking habit (p = 0.025), ECOG PS (p = 0.001), history of head and neck cancers (p = 0.017), prior neck RT (p = 0.009), anemia (p = 0.023), and operative time (p = 0.002) were significantly associated with postoperative dysphagia. There was a non-significant trend toward significance postoperative dysphagia and PNI (p = 0.075) and tumor size (p = 0.068) (Table 2).

**Table 2:** Univariate analysis.

Variables		Swallowing functions (FOSS stage 0) N = 53	Swallowing functions (FOSS stage 1–5) N = 31	p-value
**Age (years)**		64 (46–85)	69 (40–87)	0.015
	<80	52 (98.1%)	25 (80.6%)	0.009
	≥80	1 (1.9%)	6 (19.4%)	
**Sex**	Male	49 (92.5%)	29 (93.5%)	1.000
	Female	4 (7.5%)	2 (6.5%)	
**BMI (kg/m** ^**2**^ **)**	≥18.5	43 (81.1%)	24 (77.4%)	0.780
	<18.5	10 (18.9%)	7 (22.6%)	
**Primary site**	Oropharynx	11 (20.8%)	7 (22.6%)	0.827
	Hypopharynx	40 (75.5%)	22 (71.0%)	
	Larynx	2 (3.8%)	2 (6.5%)	
**Smoking habit**	Never/Former	34 (64.2%)	27 (87.1%)	0.025
	Current	19 (35.8%)	4 (12.9%)	
**Alcohol drinking**	Never/Former	9 (17.0%)	8 (25.8%)	0.402
	Current	44 (83.0%)	23 (74.2%)	
**ECOG PS**	0	52 (98.1%)	23 (74.2%)	0.001
	1	1 (1.9%)	8 (25.8%)	
**History of head and neck cancer**	Yes	31 (58.5%)	26 (83.9%)	0.017
	No	22 (41.5%)	5 (16.1%)	
**History of esophageal cancer**	Yes	42 (79.2%)	24 (77.4%)	1.000
	No	11 (20.8%)	7 (22.6%)	
**Prior neck radiation therapy**	Yes	8 (15.1%)	13 (41.9%)	0.009
	No	45 (84.9%)	18 (58.1%)	
**Hemoglobin (g/dL)**		13.8 (10.2–16.8)	12.5 (10.5–15.3)	0.107
	Anemia	30 (56.6%)	9 (29.0%)	0.023
	Non-anemia	23 (43.4%)	22 (71.0%)	
**Albumin (g/dL)**		4.20 (2.9–5.2)	4.0 (3.1–5.0)	0.194
	<3.5	2 (3.8%)	4 (12.9%)	0.190
	≥3.5	50 (96.2%)	27 (87.1%)	
**NLR**		2.10 (0.63–6.08)	2.50 (0.81–4.99)	0.499
	<2.1	25 (47.2%)	11 (35.5%)	0.364
	≥2.1	28 (52.8%)	20 (64.5%)	
**PLR**		162.6 (32.7–396.4)	161.5 (53.2–472.5)	0.197
	<237	44 (83.0%)	22 (71.0%)	0.271
	≥237	9 (17.0%)	9 (29.0%)	
**PNI**		48.8 (33.6–63.9)	47.1 (38.9–55.6)	0.090
	<44.5	6 (11.5%)	9 (29.0%)	0.075
	≥44.5	46 (88.55%)	22 (71.0%)	
**Histology**	*In situ*	28 (52.8)	12 (38.7)	0.260
	SCC	25 (47.2)	19 (61.3)	
**pT**	pTis	28 (52.8%)	12 (38.7%)	0.320
	pT1	17 (32.1%)	16 (51.6%)	
	pT2	7 (13.2%)	3 (9.7%)	
	pT3	1 (1.9%)	0	
**Ly**	Negative	52 (98.1%)	30 (96.8%)	1.000
	Positive	1 (1.9%)	1 (3.2%)	
**V**	Negative	50 (94.3%)	27 (87.1%)	0.415
	Positive	3 (5.7%)	4 (12.9%)	
**Tumor size (mm** ^**2**^ **)**		300 (14.0–2075.0)	460 (25.0–2500.0)	0.116
	<323	27 (50.9%)	9 (29.0%)	0.068
	≥323	26 (49.1%)	22 (71.0%)	
**Period from discovery to surgery (day)**		75 (0–998)	60.0 (10–287)	0.241
**Operative time (min)**		69 (13–180)	104 (20–600)	0.007
	<103	42 (79.2)	14 (45.2)	0.002
	≥103	11 (20.8)	17 (54.8)	

FOSS, Functional Outcome Swallowing Scale; BMI, body mass index; ECOG PS, Eastern Cooperative Oncology Group Performance Score; NLR, neutrophil/lymphocyte ratio; PLR, platelet/lymphocyte ratio; PNI, prognostic nutritional index; Ly, lymphatic invasion, V, blood vessel invasion.

Multivariate analysis identified ECOG PS (p = 0.005), prior neck RT (p = 0.012), and operative time (p = 0.012) as independent predictors for postoperative dysphagia (Table 3). The relationship between the operative results and swallow functions is presented in Table 4.

**Table 3:** Multivariate analysis.

Characteristic	Odds ratio (95% CI)	p-value
**Age (years)**		
**<80**	Reference	
**≥80**	3.196 (0.237–43.046)	0.381
**Performance status**		
**0**	Reference	
**1**	41.714 (3.173–548.345)	0.005
**History of head and neck cancer**		
**No**	Reference	
**Yes**	2.881 (0.513–16.180)	0.230
**Previous radiation on the neck**		
**No**	Reference	
**Yes**	6.285 (1.486–26.586)	0.012
**Smoking status**		
**Never/former**	Reference	
**Current**	0.262 (0.051–1.353)	0.110
**Hemoglobin**		
**Anemia**	Reference	
**Non-anemia**	2.043 (0.198–21.042)	0.548
**Operative time (min)**		
**<103**	Reference	
**≥103**	6.040 (1.485–24.556)	0.012

CI, confidence interval; PNI, prognostic nutritional index.

**Table 4:** Operative results.

Variables	Swallowing functions					
		Stage 0 N = 53	Stage 1 N = 15	Stage 2 N = 8	Stage 3–5 N = 8	p-value
**SICU stay (days)**		2 (0–7)	3 (0–6)	3.5 (2–4)	4 (0–10)	0.235
**Hospitalization (days)**		11 (5–25)	12 (6–33)	16 (8–46)	34.5 (8–93)	0.010
**Pneumonia**	Yes	3 (5.7%)	6 (40.0%)	2 (25.0%)	4 (50.0%)	0.001
	No	50 (94.3%)	9 (60.0%)	6 (75.0%)	4 (50.0%)	
**Overall dysphonia Grade**	Unknown	2 (3.8%)	1 (6.7%)	0	1 (12.5%)	0.214
	0	48 (90.6%)	12 (80.0%)	5 (62.5%)	5 (62.5%)	
	1	3 (5.7%)	2 (13.3%)	3 (37.5%)	2 (25.0%)	
**Recurrence**	Yes	3 (5.7%)	0	2 (25.0%)	0	0.084
	No	50 (94.3%)	15 (100%)	6 (75.0%)	8 (100%)	
**Cancer-specific death**	Yes	0	0	1 (12.5%)	0	0.022
	No	53 (100%)	15(100%)	7 (87.5%)	8 (100%)	

SICU, surgical intensive care unit.

There was an association between hospitalization and impaired swallowing function (p = 0.010). Patients who contracted pneumonia comprised 5.7%, 40%, 25%, and 50% of patients with FOSS stages 0, 1, 2, and 3, respectively, indicating a significant association with swallowing impairment (p = 0.001). There were no significant associations with surgical intensive care unit stay, overall dysphonia grade recurrence, cancer-specific death, or swallowing function.

## Discussion

We investigated the factors related to swallowing function in patients who underwent TOS for laryngopharyngeal cancers. Outcome variables were binarized as FOSS stages 0 and 1–5, because there was a significant difference in postoperative pneumonia between FOSS stages 0 and 1–5. The results of the present study clarified some factors that may help predict postoperative dysphagia in patients who underwent TOS.

Univariate analysis showed that age was significantly correlated with postoperative dysphagia. This contrasts with previous reports stating that age at the time of surgery is not associated with permanent swallowing dysfunction [8,9]. Tomifuji et al. reported that age at the time of surgery was not statistically significant, because they performed transoral videolaryngoscopic surgery (TOVS) for selected patients in their 80s [8]. However, patients older than 65 years have been reported to have a significantly higher number of postoperative gastrostomies [10].

We performed TOS for patients aged ≥18 years at the time of resection. Six (85.7%) of seven patients who were ≥80 years of age had postoperative dysphagia. As the number of TOSs in older adults is likely to increase in the future, further studies are needed to assess the risk of postoperative swallowing impairment in this age group.

We found that current smokers had significantly more dysphagia than never-smokers and former smokers. It is well-established that smokers are at a significantly higher risk than non-smokers for postoperative complications, and we need to strongly advise patients to quit smoking after TOS.

The phenomenon of field cancerization in the upper gastrointestinal tract is a well-known process of transformation of an existing precancerous lesion into a malignancy [11]. The clinical concept was formulated to explain the carcinogenesis of multiple cancers and precursor lesions in the upper gastrointestinal tract.

A history of head and neck cancers or esophageal cancer seems to be an important factor for postoperative swallowing function. Patients received therapy related to swallowing function for head and neck cancer or esophageal cancer before TOS. Univariate analysis showed that a history of head and neck cancer was significantly associated with postoperative dysphagia, but a history of esophageal cancer was not. TOS-eligible patients are often diagnosed using esophagogastroduodenoscopy following esophageal cancer assessment, which might account for the 66 (78.6%) patients in this study who had a history of esophageal cancer.

It has been reported that abstinence from alcohol and smoking is required to prevent the occurrence of a second primary or multiple SCCs in the upper gastrointestinal tract associated with field cancerization [12]. These findings affirm the benefits of alcohol and smoking abstinence, which may include the decrease in postoperative dysphagia among patients who underwent TOS.

Multivariate analysis identified ECOG PS, prior neck RT, and operative time as independent predictors of postoperative dysphagia. Eligible patients had an ECOG PS of 0–1: 75 patients had PS 0 and 9 patients had PS 1. Although there were no cases of PS 2 or above, PS 1 was associated with significantly more dysphagia than PS 0. Hence, the preoperative condition of a patient might play a role in postoperative swallowing impairment.

Due to the widespread use of NBI by magnifying endoscopy, tumor recurrence or appearance of new lesions within the irradiation field following chemoradiotherapy or radiotherapy therapy are more easily detected. A previous study reported that NBI is an excellent observation method for follow-up screening of patients with laryngeal and hypopharyngeal cancers treated with radiotherapy or chemoradiotherapy [13]. In this study, 21 (25.0%) patients had a history of neck irradiation. Prior neck RT was significantly associated with postoperative dysphagia.

Fujiwara et al. [14] reported that soft tissue fibrosis and sclerosis caused by radiotherapy adversely affects laryngeal suspension. Inflammation and fibrosis, which accompany irradiation, can also alter muscle and nerve electrophysiology, resulting in swallowing impairment.

Tomifuji et al. [15] performed salvage TOVS after radiotherapy or chemoradiotherapy and reported that swallowing function was severely impaired, leading to infection and necrosis of the wound. Furthermore, it was reported that if salvage ELPS is performed after RT, patients should be followed up on an outpatient basis to monitor the onset of subsequent cancers. Postoperative management should be performed cautiously [16].

Operative time was associated with postoperative swallowing function. There are reports that a large resection area is associated with postoperative dysphagia [15] and that advanced T-stage tumors are associated with poor postoperative swallowing function [9,10].

In this study, T stage and tumor size were not associated with poor swallowing function after ELPS. However, there were few patients with advanced T stages in this study. Although there was a trend toward postoperative dysphagia in terms of tumor size, the difference was not statistically significant. Factors associated with operative time were examined and correlated with a history of head and neck cancer and tumor size (p = 0.014, p = 0.022). This result suggests that tumor size may be associated with postoperative swallowing function but may not have been an associated factor due to the presence of small tumor diameter lesions with a history of head and neck cancer. In other words, tumor size and difficulty of resection were associated with operative time, and a longer operative time was assumed to be associated with dysphagia. However, further studies are needed, especially in many patients with an advanced T stage.

The pyriform sinus and arytenoid are important areas for postoperative swallowing function.

Several studies have found that arytenoid or pyriform sinus resection are associated with dysphagia [8,17].

Although 51 (82.3%) of 62 cases of hypopharyngeal carcinoma were in the pyriform sinus, the primary site was not a significant factor in this study. Other studies found that arytenoid resection was associated with dysphagia [8,17]. However, in this study, no patients underwent arytenoid resection.

In conclusion, we evaluated the factors related to swallowing function in patients who underwent TOS for laryngopharyngeal cancers. Multivariate analysis identified ECOG PS, prior neck RT, and operative time as independent predictors of postoperative dysphagia. This study suggests that patients with poor PS or those who received prior neck RT have a detailed preoperative evaluation of swallowing function, in addition to a brief swallowing evaluation, including the usual interview. In the future, we would like to develop criteria for preoperative swallowing evaluation and create a system that can identify patients who are suitable for TOS.
